# Asthma Hospitalizations in Children Before and After COVID-19: Insights from Northern Colombia

**DOI:** 10.3390/clinpract15100184

**Published:** 2025-10-06

**Authors:** Moisés Árquez-Mendoza, Karen Franco-Valencia, Marco Anaya-Romero, Maria Acevedo-Cerchiaro, Stacey Fragozo-Messino, Deiby Luz Pertuz-Guzman, Jaime Luna-Carrascal

**Affiliations:** 1 Life Science Research Center, Facultad de Ciencias Básicas y Biomédicas, Universidad Simón Bolívar, Barranquilla 080002, Colombia; moises.arquez@unisimon.edu.co (M.Á.-M.); karen.franco@unisimon.edu.co (K.F.-V.); marco.anaya@unisimon.edu.co (M.A.-R.); 2 Facultad de Ciencias de la Salud, Universidad Simón Bolívar, Barranquilla 080002, Colombia; marianurys@hotmail.com (M.A.-C.); staceyfragozo@gmail.com (S.F.-M.); 3 Grupo de Investigaciones Microbiologicas y Biomedicas de Cordoba (Gimbic), Universidad de Córdoba, Montería 230002, Colombia; deibypertuz@correo.unicordoba.edu.co

**Keywords:** asthma, children, COVID-19, environmental influences

## Abstract

**Background:** Pediatric asthma is a multifactorial condition influenced by environmental, biological, and social determinants. The COVID-19 pandemic introduced new variables that may have affected the severity and management of asthma in children and adolescents, particularly through changes in healthcare access, treatment adherence, and exposure to environmental risk factors. **Objective:** To evaluate the association between asthma severity and various factors including nutritional status, corticosteroid use, COVID-19 vaccination, and pollutant exposure before and during the COVID-19 pandemic in a pediatric population. **Methods:** A retrospective analysis was conducted using 307 medical records of patients aged 3 to 17 years. Data collected included sociodemographic characteristics, nutritional indicators, history of corticosteroid use, vaccination status against COVID-19, and exposure to environmental pollutants. Asthma severity was assessed using the pulmonary score, and multiple statistical analyses, including logistic regression using the Bayesian Logistic Regression Model (BLRM), were employed to identify significant associations. **Results:** The analysis revealed a statistically significant impact of the pandemic on hospitalization rates (*p* = 0.0187) and the use of corticosteroids (*p* = 0.009), indicating changes in asthma management during this period. Notable differences were observed in the geographic distribution of mild versus severe asthma cases prior to the pandemic, associated with nutritional status and gender (*p* = 0.018). During the pandemic, breastfeeding history, body weight, and hospitalization emerged as significant predictors of asthma severity (*p* < 0.05). In addition, breastfeeding in young children (aged 3 to 6 years) and hospitalization were strongly associated with pulmonary scores, with significance values of 0.022 and 0.012, respectively, as identified by the BLRM. **Conclusions:** These findings suggest that the pandemic context influenced both the clinical course and management of pediatric asthma. Preventive strategies should consider individual and environmental factors such as nutrition, early-life health practices (e.g., breastfeeding), and equitable access to appropriate asthma care and vaccination. Tailoring pediatric asthma management to these variables may improve outcomes and reduce disparities in disease severity.

## 1. Introduction

Asthma is one of the most prevalent chronic respiratory diseases worldwide, as well as a leading cause of medical consultations among children and adolescents [1,2]. It affects over 250 million people globally, with many cases being diagnosed in childhood, making asthma a significant public health issue [3]. In 2019 alone, asthma affected 262 million people and was responsible for approximately 461,000 deaths [4].

The development and progression of asthma are influenced by multiple components of an individual’s exposome, especially the interaction between genetic predisposition and environmental exposure. This interaction can affect disease severity and prognosis [5]. Asthma is also closely linked to allergic diseases. Viral respiratory infections can exacerbate allergic responses and trigger asthma attacks [6]. The World Allergy Organization estimates that 40% of the global population suffers from allergic conditions, with comparable prevalence rates in tropical and temperate regions [7].

In Colombia, the prevalence of asthma symptoms increased from 10.4% to 12% over the past decade. Significant variations were observed across different age groups and geographical regions [8]. Other studies have reported asthma prevalence rates among children and adolescents ranging from 8.8% to 30.8% [9].

Barranquilla, the capital of Colombia’s Caribbean region and the Atlántico Department, has a complex environmental context that negatively impacts pediatric respiratory health due to the interplay of urban, peri-urban, and rural factors. In the urban core, vehicular traffic and industrial processes are the main sources of air pollution, emitting pollutants such as PM_2_._5_, PM_10_, NOx, CO, and SO_2_ [10]. Seasonal forest fires in the nearby Isla Salamanca Natural Park produce episodic spikes in particulate matter that exacerbate pediatric asthma (MDPI). In peri-urban and rural areas, agricultural emissions, biomass burning, dust from unpaved roads, and indoor pollution from biomass fuel use further elevate the risk of respiratory infections and childhood asthma [11]. Although urban children are more exposed to traffic-related pollutants, children in rural communities often have worse respiratory outcomes due to limited access to healthcare and asthma control measures. This disparity, coupled with Barranquilla’s tropical climate marked by high temperatures, humidity, and seasonal rainfall, creates a multifaceted environmental scenario that likely increases the incidence and severity of childhood asthma in the region [12].

Understanding the epidemiological patterns of asthma is crucial, especially in pediatric populations, due to the significant clinical impact of asthma and its strong links to environmental and allergic factors. This study aimed to describe the clinical and epidemiological characteristics of children and adolescents hospitalized for asthma exacerbations in a tertiary pediatric hospital in Barranquilla, Colombia, during three time periods: before, during, and after the pandemic. This study aimed to identify factors associated with asthma severity and hospitalization, as well as to inform the development of more effective prevention strategies.

## 2. Materials and Methods

### 2.1. Study Area and General Description of the Population

The study was conducted at a tertiary pediatric hospital in Barranquilla, Colombia, a coastal city in the Caribbean region with a dry tropical climate. The city has an annual average temperature ranging from 27 to 29 °C, with distinct dry (December–April) and rainy (May–November) seasons. These periods are interrupted by the veranillo de San Juan in June and July. The average annual rainfall is 821 mm, with peak humidity in September and November. These conditions are influenced by trade winds and the Intertropical Convergence Zone [13]. These climatic features affect thermal comfort, air quality, and the dispersion of atmospheric and biological pollutants, such as fungal spores and allergens. These factors are particularly relevant in pediatric populations because their respiratory and immune systems are immature, increasing their susceptibility to inhaling and retaining fine particles and airborne pathogens [14].

According to the epidemiological profile and population characterization of the city of Barranquilla for the year 2023, in relation to respiratory diseases, 5295 cases were reported in children aged 0 to 5 years, 2823 cases in the 6 to 11 age group, and 2059 cases in adolescents aged 12 to 17 years [15]. Patients come from the city of Barranquilla and other municipalities in its metropolitan area, as shown by the geographic distribution see Supplementary Table S1.

The initial study population consisted of 6024 children and adolescents, aged 3 to 17, who were admitted to Barranquilla Pediatric Hospital between 2019 and 2023. Of those, 307 patients were selected based on the following inclusion criteria: (1) confirmed asthma diagnosis; (2) documentation of a pulmonary score in the medical record; (3) presentation to the emergency department due to an asthma exacerbation; (4) subsequent hospitalization. Exclusion criteria included a history of liver disease or renal insufficiency, incomplete medical records, and age under three years.

After applying these criteria, 307 eligible patients were identified and categorized into three independent study groups based on their date of admission to the hospital. The pre-pandemic group included 87 children admitted between 1 January 2019, and 29 January 2020. The pandemic group included 175 children admitted between 30 January 2020, and 5 May 2023. The post-pandemic group included patients admitted between 6 May 2020, and 31 December 2023. Thus, the analysis of the clinical cases was conducted during the period officially declared the global pandemic of SARS-CoV-2. In Colombia, the health emergency was declared on 12 March 2020, through Resolution 385 [16], issued by the Ministry of Health and Social Protection. The health emergency was officially concluded on 30 June 2022, in accordance with Resolution 666 of 2022 [17].

In parallel, the Colombian pediatric population was progressively incorporated into the National COVD-19 Vaccination Program. On 31 October 2021, the program extended vaccination to children aged 3–11 years with the inactivated virus vaccine Sinovac–CoronaVac. Between late 2021 and early 2022, adolescents aged 12–17 years were included with mRNA vaccines (Pfizer-BioNTech and Moderna Norwood, Massachusetts (EE. UU.)). By mid-2022, booster doses were authorized for adolescents, and subsequent recommendations prioritized children with underlying conditions, such as asthma or immunosuppression. In 2023, Resolution 986 established updated technical and operational guidelines for vaccination across all age groups. This was followed by Resolution 1862, which incorporated additional pediatric formulations, such as Spikevax (Moderna Pediatric), for children aged 6 months to 2 years [18,19,20].

It is important to note that the pulmonary score is an internationally recognized clinical tool used to evaluate the severity of asthma exacerbations in children and adolescents. The score is based on three core clinical signs—respiratory rate, use of accessory muscles, and wheezing on auscultation—each of which is scored from 0 to 3 [21].

The total score ranges from 0 to 9 and categorizes exacerbations as mild (0–3), moderate (4–6), or severe (7–9). This scoring system has been widely adopted in pediatric asthma management protocols to guide treatment decisions and hospitalization criteria [22].

### 2.2. Statistical Analysis

Data was compiled into Excel matrices using variables extracted from medical records. To ensure confidentiality, each patient was assigned a double-blind identification code. the analysis included sociodemographic variables (sex and age) and clinical variables, including the patient’s nutritional status, which can be determined by the presence or absence of malnutrition and by categorizing the patient’s weight in relation to his or her height as underweight, adequate, overweight, or obese; breastfeeding history; pulmonary score; corticosteroid use; COVID-19; vaccine administration status; and exposure to environmental pollutants. The pulmonary score was the primary response variable, categorized as mild (0–3), moderate (4–6), or severe (7–9), according to established asthma severity criteria.

In the initial statistical analysis, patients were classified into three groups based on their period of hospitalization: pre-pandemic, pandemic, or post-pandemic. Within each period, children were categorized into two age groups: preschool (ages 3–6) and older (ages 7–17). The dataset included qualitative and quantitative variables. Contingency table analyses were performed using the chi-squared test (χ^2^) for quantitative variables and Fisher’s exact test for categorical variables, with a significance level of *p* < 0.05.

Because there was a low number of cases after the pandemic, patients were grouped into two subgroups based on their pulmonary score. Subgroup 1 (the pre-pandemic group) included individuals with mild (0–3) and moderate/severe (4–9) scores. Subgroup 2 included individuals with the same score range while maintaining the prespecified age range. Binary logistic regression models (BLRM) using maximum likelihood estimation were applied to assess associations, with a significance threshold of *p* < 0.05. the data of the analyses performed are available. See Supplementary Table S1.

## 3. Results

During the pre-pandemic period, there were no statistically significant differences in the concentration of hospitalized and non-hospitalized cases in Barranquilla. However, during the COVID-19 emergency, a significant association was observed between geographic location and hospitalization (*p* = 0.008). There was a notable increase in hospitalizations in Barranquilla (38.28%) and its metropolitan area (33.14%). Regarding gender, males consistently represented a higher proportion of cases across all periods. A statistically significant difference was observed during the emergency phase (*p* = 0.003), and a particularly pronounced male predominance was seen in the post-COVID period (75.55%). In terms of age distribution, preschool children (ages 3–6) made up the majority of patients in every period. However, there was a slight increase in adolescents (ages 7–17) during the post-COVID stage (22.22%). While this shift was not statistically significant (*p* = 0.091), see Table 1 for more information.

The analysis of clinical and environmental variables throughout the three study periods revealed relevant trends. Regarding breastfeeding, no significant differences were observed in its prevalence among preschool children (ages 3–6) and older children (ages 7–17) during any of the study periods. The proportions remained relatively stable, and there was no statistical association (*p* = 0.078 during the COVID-19 emergency). However, corticosteroid use increased significantly during the pandemic, particularly during the emergency phase when 50.28% of participants received this treatment, compared to 34.48% in the pre-pandemic period. This difference was statistically significant (*p* = 0.009), and the trend continued in the post-COVID period, as seen in Table 2.

We also analyzed the COVD-19 vaccination schedule over time. While no children were vaccinated before the pandemic, 26.85% received the vaccine during the emergency phase. This proportion increased to 44.44% in the post-COVID period. The difference was highly significant (*p* = 0.000). The proportion of individuals exposed to environmental contaminants decreased significantly during and after the pandemic (from 48.27% in the pre-pandemic period to 29.71% during the pandemic and 31.11% afterward; *p* = 0.010), as seen in Table 2.

During the pre-pandemic period, the nutritional status of both teen girls and teen boys was mostly adequate, although a higher percentage of boys were overweight or obese. This difference was statistically significant for overweight individuals (*p* = 0.018). During the COVID-19 emergency, cases of malnutrition and obesity increased in both sexes, and the proportion of individuals with adequate nutritional status decreased slightly. However, the differences by sex did not reach statistical significance (*p* = 0.303). In the post-COVID period, the distribution showed an even more pronounced pattern; teen girls were overweight or obese, while 8.88% of boys were overweight and 6.66% were obese, though the difference was not significant (*p* = 0.612), as can be seen Table 3.

Regarding the pulmonary score, a higher frequency of mild cases was observed in both sexes throughout the study, with a clear predominance of mild scores in boys, particularly in the post-COVID stage (68.88% vs. 22.22% teen girls). Moderate and severe cases were rare and distributed similarly between the sexes in all study phases (*p* > 0.05 in all periods), as can be seen in Table 3.

### 3.1. Observed Patterns of Asthma Severity in Individual Groups Before and After the Pandemic

**Figure 1:**
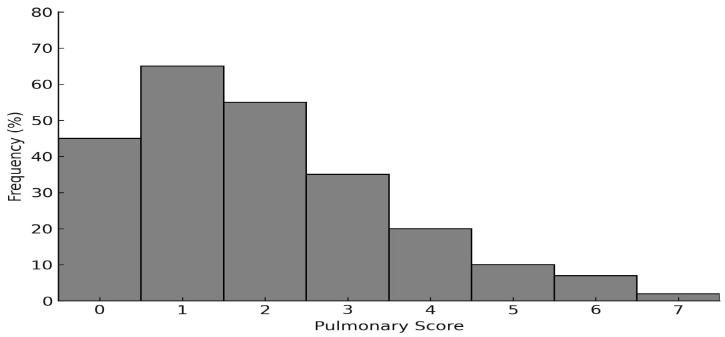
Frequency distribution of pulmonary scores among asthmatic patients during the pre-pandemic period.

**Figure 2:**
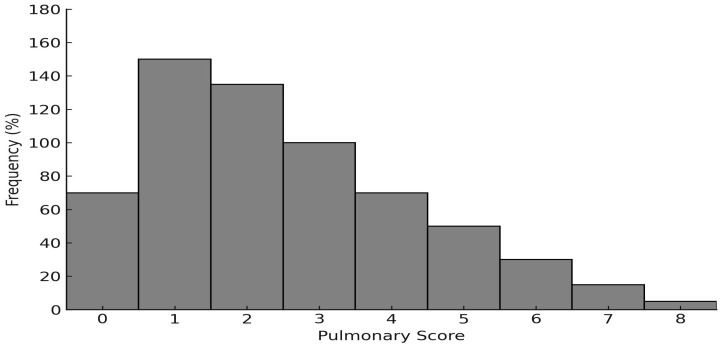
Frequency distribution of pulmonary scores among asthmatic patients during the COVID-19 pandemic and post-pandemic periods.

Throughout the pre-pandemic period, the general population experienced an upward trend in reported cases of mild, moderate, and severe asthma, as illustrated in Figure 1. Figure 2, in contrast, shows a higher proportion of cases classified as mild or moderate asthma and a marked decline in severe cases during the post-pandemic period.

#### 3.1.1. Binary Logistic Regression Model Analysis—Pre-Pandemic, Subgroup 1

The binary logistic regression model accounts for approximately 10.9% of the variability in the classification of mild versus moderate cases. The odds ratio indicates that the model as a whole is not statistically significant. Additionally, variables such as sex, age, weight, and breastfeeding do not show a meaningful impact on the prediction, as can be seen in Table 4.

#### 3.1.2. Binary Logistic Regression Model Analysis Pandemic, Subgroup 2

The binary logistic regression model explained approximately 89.1% of the mild and moderate/severe cases. In addition, breastfeeding in young children (aged 3 to 6 years) and hospitalization were the variables most strongly associated with pulmonary scores, with significance values of 0.022 and 0.012, respectively. In contrast, sex and age did not contribute significantly to the model, as can be seen in Table 5.

## 4. Discussion

This study provides a comprehensive overview of the clinical/sociodemographic, and epidemiological characteristics associated with asthma as a leading cause of hospitalization among children and adolescents at a tertiary pediatric center in Barranquilla, Colombia. The findings offer insights into how various clinical, social, and environmental factors influence the development, severity, and management of pediatric asthma.

One of the most notable findings was the variation in asthma-related hospitalization rates observed during different study periods. Geographic differences observed during the pandemic’s emergence suggest that it may have affected the incidence and clinical management of asthma exacerbations locally [23]. Preliminary post-pandemic studies suggest that the Delta and Omicron variants led to increased hospitalizations in children and adolescents [24], although there is no evidence of increased mortality in this population [25]. In Colombia, the Delta variant became predominant between June and September 2021, corresponding to the third wave of the pandemic, while the Omicron variant, introduced in December 2021, was responsible for the fourth wave (January–March 2022), which showed a marked rise in case incidence but a comparatively lower lethality due to vaccination coverage [26]. Environmental factors and climatic variations typical of tropical countries could further influence the increase in mild-to-moderate asthma cases in our region [27]. This increase may be partially explained by the rise in viral respiratory infections during the pandemic. Previous research has demonstrated that viruses, particularly respiratory syncytial virus (RSV), increase the risk of asthma development and symptom exacerbation [28].

Nutritional status also emerged as a key factor, with notable gender-specific differences, as shown in Table 3. Notably, males exhibited more pronounced variations. Being overweight or obese has been linked to a higher risk of asthma exacerbations [29] and reduced responsiveness to certain treatments [30]. Conversely, undernutrition may impair immune and pulmonary function, thereby exacerbating asthma symptoms [31]. These findings underscore the importance of incorporating nutritional assessment and intervention into comprehensive pediatric asthma care.

Obesity and undernutrition, at opposite ends of the malnutrition spectrum, can influence asthma severity and disease control. Systemic inflammation may be promoted by obesity and the response to standard therapies may be blunted as a result [32]. Meanwhile, infections and complications are more likely to occur in individuals who are undernourished. These effects are likely amplified in socioeconomically vulnerable settings, such as food-insecure households in Colombia’s Caribbean region [33].

During the pandemic, several adjustments were incorporated into pediatric asthma management protocols. The main changes included replacing nebulized bronchodilators with metered-dose inhalers and spacers to reduce aerosol generation, maintaining inhaled corticosteroid therapy without interruption, and prioritizing remote consultations to ensure follow-up and adherence. In hospital settings, systemic corticosteroids and controlled oxygen therapy were preferred while minimizing aerosol-generating procedures. Furthermore, vaccination strategies against influenza and COVID-19 were reinforced for children with asthma [34]. These adaptations aimed to minimize the risk of viral transmission while maintaining adequate asthma control.

Nevertheless, particular attention should be given to the use of inhalers in adolescent populations, where a transition toward self-management is expected but adherence often remains inconsistent. Previous studies in Latin America have shown that asthma self-management among adolescents continues to be suboptimal, with a high reliance on caregivers for medication administration and medical decision-making [35]. Evidence also suggests that female adolescents may experience more severe symptoms and exacerbations, potentially due to hormonal influences and psychosocial factors, which further emphasizes the need for structured support in asthma self-management [36].

The overlap of asthma and SARS-CoV-2 infection may have prompted more aggressive corticosteroid treatment to control inflammation and prevent adverse outcomes [37]. Nevertheless, balancing the benefits and risks of corticosteroid use remains essential, especially during viral outbreaks, and aligning international clinical guidelines is crucial [38,39].

Beyond clinical factors, the broader social and environmental context of Barranquilla—Colombia’s most important Caribbean coastal city—may also influence asthma outcomes. Rapid industrial expansion has contributed to increased pollution [40], which may be driving the rising prevalence and severity of asthma among children and adolescents [41,42]. Regarding the impact of COVID-19 on asthma, it is difficult to establish a direct causal relationship. Our findings suggest that the pandemic context influenced the clinical presentation and management of pediatric asthma, possibly imitating or amplifying preexisting patterns rather than initiating entirely new ones. Subsequently, local cases in Barranquilla and across Latin America should be examined with caution, including incidence and prevalence rates.

Epidemiological studies using binary logistic regression models have shown that variables such as sex, age, race, household poverty, and family structure, when adjusted for residential location, can help explain the rising burden of asthma in younger populations. This underscores the importance of localized public health strategies that address structural determinants of health. One approach is to implement systems that monitor severe asthma cases from hospital discharge through home-based care [43].

Another relevant factor in our findings was vaccination coverage. Asthma cases were more frequently observed in children and adolescents who had received all recommended vaccinations. Although concerns were initially raised about the safety of the vaccines [44] for individuals with allergies, current evidence supports their safety and tolerability for patients with asthma [45]. Furthermore, vaccination may contribute to better asthma control by reducing the risk of respiratory infections [46].

Applying the binary regression model to subgroups 1 and 2 provided a better description of moderate and severe asthma cases in the pre- and post-pandemic periods, revealing an increase in hospitalizations. A significant proportion of children were prone to severe asthma, with key variables—including breastfeeding, weight, and previous hospitalization—having a meaningful impact on pulmonary scoring. This would be related to what has been studied by considering where risk factors such as other diseases and even environmental risk factors could increase the number of cases in patients with this type of disease [47]

Breastfeeding has been associated with a lower likelihood of developing moderate-to-severe asthma, which supports its protective role against respiratory conditions in early childhood. This finding aligns with extensive literature detailing the advantages of breastfeeding for psychophysical development, nutrition, and immune system strengthening in children [48,49]. However, our results should be interpreted with caution because other uncontrolled factors in this study may have influenced the observed association.

These patterns may be explained by lifestyle changes, reduced access to healthcare, and shifts in environmental exposure due to pandemic lockdowns. As such, the findings reinforce the importance of accounting for the broader epidemiological context when designing asthma prevention and treatment strategies. The contrast between the pre-pandemic and COVID-19 emergency periods suggests that the pandemic not only altered risk exposure but may also have contributed to both the escalation and potential mitigation of pediatric asthma.

## 5. Conclusions

This study highlights the multifactorial nature of asthma in children and adolescents, emphasizing the influence of clinical, nutritional, environmental, and socioeconomic variables on disease severity and hospitalization. The COVID-19 pandemic served as a key inflection point, exposing shifts in healthcare access, treatment approaches, and risk exposure that impacted asthma outcomes.

Findings underscore the protective role of breastfeeding, the significance of nutritional status, and the implications of prior hospitalization in determining asthma severity. While corticosteroids remain central to asthma management, their use must be carefully weighed, especially during viral outbreaks such as COVID-19. Additionally, the observed association between up-to-date vaccination schedules and asthma cases supports the safety and potential benefit of immunization in respiratory disease control.

Environmental and structural factors, particularly in rapidly urbanizing regions like Barranquilla, must be integrated into public health strategies. Interventions should prioritize targeted, community-based approaches that address food insecurity, pollution, and healthcare inequities. Continued monitoring and research are essential to inform policies and ensure effective, context-specific asthma management for pediatric populations in similar settings.

## Figures and Tables

**Table 1:** Sociodemographic variables and geographic distribution of patients in the pre-pandemic, emergency COVID 19, and emergency post-COVID periods.

Period	Geographic Distribution/Total Patients	Hospitalization n (%)	Hospitalization n (%)	X^2^
Pre-pandemic		Yes	No	
	Barranquilla	23 (26.43)	34 (39.08)	
	Metropolitan area	7(8.04)	23 (26.43)	
	Total patients = 87			
Emergency COVID-19		Yes	No	
	Barranquilla	67 (38.28)	37 (21.14)	* 0.008
	Metropolitan area	58 (33.14)	13 (7.42)	
	Total patients = 175			
Emergency post-COVID		Yes	No	
	Barranquilla	17 (37.77)	17 (37.77)	
	Metropolitan area	10 (22.22)	1 (2.22)	
	Total patients = 45			
Period	Variable	Female n (%)	Male n (%)	X^2^
Pre-pandemic		32 (36.78)	55 (63.21)	
Emergency COVID-19	Gender	79 (45.14)	96 (54.85)	* 0.003
Emergency post-COVID		11 (24.44)	34 (75.55)	
Period	Variable	Preschool children 3–6 years n (%)	Children and adolescents 7–17 years n (%)	X^2^
Pre-pandemic		77 (88.50)	10 (11.49)	
Emergency COVID-19	Population/Age	157 (89.71)	18 (10.28)	0.091
Emergency post-COVID		35 (77.77)	10 (22.22)	

**Statistical test** *X*^2^. * *p* < 0.05. Significant differences.

**Table 2:** Clinical variables, maternal infants, corticosteroid use, COVID-19 vaccine, exposure to contaminants in three pre-pandemic periods, COVID-19 emergence, and post-COVID emergence.

	Breastfed Infants			Use of Corticosteroids			COVID-19 Vaccine			Exposure to Contaminants			
Period	Preschool Children 3–6 Years n (%)	Children and Adolescents 7–17 Years n (%)	X2	Yes n (%)	No n (%)	X2	Yes n (%)	No n (%)	X2	Yes n (%)	No n (%)	X2	Total Population n (%)
Pre-pandemic	34 (39.08)	53 (60.91)		30 (34.48)	57 (65.51)		0 (0)	87 (100)		42 (48.27)	45 (51.72)		87
Emergency COVID-19	67 (38.28)	108 (61.71)	0.078	88 (50.28)	87 (49.71)	* 0.009	47 (26.85)	128 (73.14)	* 0.000	52 (29.71)	123 (70.28)	* 0.010	175
Emergency post-COVID	25 (55.55)	20 (44.44)		27 (60)	18 (40)		20 (44.44)	25 (55.55)		14 (31.11)	31 (68.88)		45

**Statistical test** *X*^2^. * *p* < 0.05. Significant differences.

**Table 3:** Clinical variables, nutritional status, and pulmonary scores in male and female patients during the pre-pandemic, emergency, and post-emergency periods.

Period	Nutritional Status	Female n (%)	Male n (%)	X^2^
Pre-pandemic	Malnutrition	0 (0)	1 (1.14)	
	Appropriate	30 (34.82)	37 (42.52)	
	Overweight	2 (2.29)	10 (11.49)	* 0.018
	Obesity	0 (0)	7 (8.04)	
Emergency COVID-19	Malnutrition	3 (1.71)	4 (2.28)	
	Appropriate	65 (37.14)	70 (40)	
	Overweight	6 (3.42)	15 (8.57)	0.303
	Obesity	7 (4)	5 (2.85)	
Emergency post-COVID	Malnutrition	0 (0)	1(2.22)	
	Appropriate	11 (24.4)	26 (57.77)	
	Overweight	0 (0)	4 (8.88)	0.612
	Obesity	0 (0)	3 (6.66)	
Period	Pulmonary score	Female n (%)	Male n (%)	X^2^
Pre-pandemic	Mild (0–3)	29 (33.33)	49 (56.32)	
	Moderate (4–6)	2 (2.29)	6 (6.89)	0.416
	Severe (7–9)	1 (1.14)	0 (0)	
	Mild (0–3)	65 (37.14)	80 (45.71)	
Emergency COVID-19	Moderate (4–6)	13(7.42)	14 (8)	0.932
	Severe (7–9)	1 (0.57)	2 (1.14)	
	Mild (0–3)	10 (22.22)	31 (68.88)	
Emergency post-COVID	Moderate (4–6)	0 (0)	3 (6.66)	0.357
	Severe (7–9)	1 (0.2)	0 (0)	

**Statistical test** *X*^2^. * *p* < 0.05. Significant differences.

**Table 4:** Logistic regression analysis for the pre-pandemic.

Variable	Pulmonary Score	R^2^ (BLRM)	*p*-Value	Log-Likelihood Ratio
Breastfeeding			>0.3	
Weight	Mild	0.1091	>0.3	0.3887
Hospitalization	Moderate/severe		0.371	
Age			>0.3	

Binary logistic regression model (BLRM). Log-likelihood ratio, *p*-value.

**Table 5:** Logistic regression analysis for the COVID-19 emergency.

Variable	Pulmonary Score	R^2^ (BLRM)	*p*-Value	Log-Likelihood Ratio
Breastfeeding			* 0.022	
Weight	Mild	0.891	0.087	* 0.0097
Hospitalization	Moderate/severe		* 0.012	
Age			0.162	

Binary logistic regression model (BLRM), * *p* < 0.05. Log-likelihood ratio, *p*-value.

## Data Availability

The data presented in this study are available on request from the corresponding author due to in case of need.

## Supplementary Materials

The following supporting information can be downloaded at: https://www.mdpi.com/article/10.3390/clinpract15100184/s1. Table S1. The qualitative and quantitative variables.

## Abbreviations

The following abbreviations are used in this manuscript:

°C	Celsius
R^2^	Coefficient of determination
COVID-19	Coronavirus Disease 2019
MDLB	Binary Logistic Regression Model
SARS-CoV-2	Severe Acute Respiratory Syndrome due to Coronavirus 2
VSR	Respiratory Syncytial Virus
