# Supplementary material for: Asthma Hospitalizations in Children Before and After COVID-19: Insights from Northern Colombia
Source: Clin Pract. 2025 Oct 6;15(10):184. doi: 10.3390/clinpract15100184 (PMC12562370; doi:10.3390/clinpract15100184)
Supplement: Supplementary file 1 [file clinpract-15-00184-s001.zip › clinpract-3803737-supplementary.pdf]

clinics and practice

Article

Asthma Hospitalizations in Children Before and After COVID-19: Insights from Northern Colombia

Moisés Árquez-Mendoza [1], Karen Franco-Valencia [1], Marco Anaya-Romero [1], Maria Acevedo-Cerchiaro [2], Stacey Fragozo-Messino [2], Deiby Luz Pertuz-Guzman [3] and Jaime Luna-Carrascal [1,*]

Supplementary material

Qualitative and quantitative variables and assignments.

Gender. Male = m. female = f.
Age/years
Geographic distribution/Barranquilla/Metropolitan area
Pulmonary score. 0; 1; 2; 3; 4; 5; 6; 7; 8; 9.
Breastfed infants. Yes = s; No = n
Body weight (Kg)
Stature (m)
Hospitalization (Yes = s. No = n)
Corticosteroide Use. (Yes = s. No = n)
COVID-19 Vaccination (Yes/No). (Yes = s. No = n)
Environmental pollution exposure. (Yes = s. No = n)
Nutritional status. Malnutrition = (d. Appropriate = a. Overweight= ob. Obesity= s)

Table S1. Qualitative and quantitative variables.

Data pre-pandemic period

Gender	Age/years	Geographic	Pulmonary score	Breastfed infants	Body weigth (Kg)	Stature (m)	Hospitalization	CorticosteroidsUse	COVID-19 Vaccination	Environmental pollution exposure	Nutritional status
f	3	barranquilla	2	n	14.5	0.96	n	n	n	s	a
m	3	soledad	3	s	16.6	1.02	n	n	n	s	a
f	10	barranquilla	5	n	25	1.32	s	n	n	s	a
f	7	soledad	2	s	23	1.15	n	s	n	n	a
m	6	barranquilla	4	n	21.5	1.17	n	n	n	n	ob
m	5	barranquilla	3	s	19.2	1.02	n	n	n	n	s
m	3	soledad	3	n	18	1.06	n	n	n	n	a
f	12	palmar de varela	2	s	41	1.48	n	s	n	s	a
m	6	barranquilla	3	n	39.5	1.32	n	n	n	n	ob
m	7	baranoa	2	n	21	1.2	n	n	n	n	a
m	5	barranquilla	3	n	15.3	1.06	n	n	n	s	a
f	3	puerto colombia	1	n	14	0.95	n	s	n	n	a
m	4	barranquilla	4	s	21	1.07	n	n	n	s	s
m	4	barranquilla	3	n	16	1.06	n	n	n	s	a
m	6	galapa	4	s	23.5	1.15	n	n	n	n	s
m	4	malambo	4	n	13.5	0.95	s	n	n	s	a
f	3	barranquilla	2	n	12	0.83	n	n	n	n	a
f	4	barranquilla	1	n	16	0.99	n	n	n	n	a
m	9	barranquilla	3	s	28	132	s	n	n	n	a
m	12	barranquilla	2	n	30.5	1.27	s	n	n	n	a
f	6	barranquilla	3	n	19.5	1.09	s	n	n	n	a
m	8	barranquilla	2	n	41	1.41	s	n	n	s	s
m	3	el guamo	2	n	14	0.93	n	s	n	s	a
m	10	barranquilla	0	s	25.5	1.31	n	n	n	n	a
m	10	malambo	1	s	39	1.44	s	n	n	n	a

Continuation **Table S1. Qualitative and quantitative variables.**

Data pre-pandemic period

Gender	Age/years	Geographic	Pulmonary score	Breast-fed infants	Body weigth (Kg)	Stature (m)	Hospitalization	Cortico-steroidsUse	COVID-19 Vaccination	Environmental pollution exposure	Nutritional status
f	6	barranquilla	2	n	18.4	1.07	s	n	n	n	s
f	4	barranquilla	1	s	16	1.05	s	n	n	s	a
f	7	barranquilla	1	n	23	1.28	n	n	n	n	a
f	3	malambo	1	n	15	100	s	n	n	s	a
m	5	soledad	1	n	22.5	1.15	n	n	n	n	a
m	7	barranquilla	1	n	26.5	1.18	s	n	n	n	s
m	4	barranquilla	1	s	24	1.08	n	n	n	n	ob
f	5	barranquilla	1	n	15	1.02	n	n	n	n	a
m	4	salamina	2	s	18	1.1	n	s	n	n	a
m	8	soledad	2	n	26	1.3	n	n	n	s	a
m	3	soledad	2	n	14	0.99	n	s	n	s	a
m	4	sabanagrande	3	n	18.5	1	n	s	n	s	s
f	4	malambo	1	s	18	1.1	s	s	n	s	a
m	4	soledad	3	s	17.5	1.09	s	n	n	s	a
f	4	soledad	9	n	17	1.09	n	s	n	s	a
m	3	barranquilla	6	n	16	0.7	n	s	n	s	ob
f		barranquilla	2	s	14.5	0.96	n	n	n	n	a
m	3	soledad	2	s	16.6	1.02	n	n	n	s	a
f	10	barranquilla	3	s	25	1.32	s	n	n	s	a
f	7	soledad	2	n	19	1.17	n	s	n	s	a
m	6	barranquilla	3	n	22	1.21	n	n	n	n	a
m	5	barranquilla	2	n	22	112	n	n	n	n	s
m	3	soledad	2	n	15	1.05	n	n	n	s	a
f	12	palmar de varela	2	n	41	1.48	n	s	n	n	a
m	6	barranquilla	2	s	39.5	1.32	n	n	n	s	ob
m	7	baranoa	2	s	21	1.2	n	n	n	n	a

Continuation **Table S1. Qualitative and quantitative variables.**

Data pre-pandemic period

Gender	Age/years	Geographic	Pulmonary score	Breastfed infants	Body weigth (Kg)	Stature (m)	Hospitalization	Corticosteroide Use	COVID-19 Vaccination	Environmental pollution exposure	Nutritional status
f	3	soledad	2	s	10	0.8	n	s	n	n	a
m	5	barranquilla	2	s	15.3	1.06	n	n	n	n	a
f	3	puerto colombia	3	n	14	0.95	n	s	n	n	a
m	4	barranquilla	3	s	17	1.07	n	n	n	s	a
m	4	barranquilla	2	s	16	1.06	n	n	n	s	a
m	6	galapa	2	s	23.5	1.15	n	n	n	n	s
f	14	barranquilla	1	n	61	1.56	n	n	n	n	s
m	5	barraquilla	3	s	16.5	109	n	n	n	n	a
m	12	barraquilla	2	s	43	1.61	n	n	n	s	a
m	9	barraquilla	3	s	37	1.25	n	n	n	s	ob
m	5	barranquilla	2	s	16.3	1.06	n	s	n	s	a
m	6	barranquilla	2	s	18.5	1.11	n	s	n	n	a
m	5	barranquilla	1	n	16	1.05	n	s	n	n	a
f	10	barraquilla	1	s	25	1.30	n	s	n	n	a
f	4	barraquilla	2	n	14.2	1	n	s	n	n	a
m	3	barranquilla	3	s	14.5	0.96	s	n	n	s	a
m	12	barranquilla	2	n	33	1.49	s	s	n	n	a
m	3	barranquilla	1	n	16	0.97	s	s	n	s	a
m	3	barranquilla	1	n	17.5	1.01	s	n	n	s	a
m	12	barranquilla	1	n	56	1.58	n	n	n	s	s
f	3	barranquilla	1	n	14	0.89	n	s	n	s	a
f	4	soledad	2	n	15.5	1.03	n	n	n	s	a
m	4	barranquilla	2	n	18	1.1	n	n	n	s	a
m	12	barranquilla	2	n	30.5	1.27	n	n	n	s	a
f	3	barranquilla	1	n	13.5	0.92	s	s	n	n	a

Continuation **Table S1. Qualitative and quantitative variables.**

Data Emergency Covid-19 period

Gender	Age/years	Geographic	Pulmonary score	Breast-fed infants	Body weigth (Kg)	Stature (m)	Hospitalization	CorticosteroidsUse	COVID-19 Vaccination	Environmental pollution exposure	Nutritional status
m	11	barranquilla	2	s	42	1.45	S	s	n	s	s
f	12	barranquilla	7	n	66	1.56	s	n	n	n	ob
m	3	malambo	1	n	12	0.87	s	s	n	n	a
m	4	malambo	1	s	17.75	0.78	n	n	n	s	ob
f	3	malambo	3	s	12.5	0.90	s	n	n	s	a
m	4	malambo	2	s	15	0.99	s	s	n	n	a
m	3	barranquilla	2	n	12	0.89	s	s	n	s	a
f	7	malambo	6	s	20	1.27	s	s	s	n	a
f	3	barranquilla	4	n	11.9	0.99	s	s	n	n	d
m	4	malambo	1	s	15	1.09	s	s	n	s	d
f	10	barranquilla	3	s	34	1.3	s	s	s	n	a
m	8	barranquilla	2	s	27	1.23	s	n	s	n	s
f	6	malambo	4	n	22	1.16	s	n	s	n	a
m	3	soledad	0	n	14	1	s	s	n	n	a
f	5	soledad	1	n	15	1	s	s	n	n	a
m	4	galapa	1	s	15	1	s	s	s	n	a
f	3	barranquilla	0	n	14	0.75	s	s	n	n	ob
m	10	barranquilla	3	n	28.5	130	n	n	n	n	a
m	5	barranquilla	5	n	21.7	116	s	n	n	n	a
f	3	barranquilla	3	n	13	0.9	n	n	n	s	a
m	12	barranquilla	5	n	64	160	s	n	n	n	ob
f	5	barranquilla	3	n	15.5	104	s	n	n	s	a
f	8	zapayán	2	n	24	118	s	n	n	n	a
f	4	barranquilla	2	n	13.5	0.98	s	n	n	n	a

Continuation **Table S1. Qualitative and quantitative variables.**

Emergency Covid-19 period

Gender	Age/years	Geographic	Pulmonary score	Breast-fed infants	Body weigth (Kg)	Stature (m)	Hospitalization	Corticoster-oidsUse	COVID-19 Vaccination	Environmental pollution exposure	Nutritional status
m	3	malambo	3	n	17	0.99	s	n	n	n	s
f	8	remolino	2	n	28.4	1.32	s	n	n	n	a
m	3	calamar	4	s	24.5	1.06	s	s	n	n	ob
f	5	baranoa	2	n	15	0.96	n	n	n	n	a
m	3	campo de la cruz	2	n	14	0.94	n	n	n	n	a
m	10	santa lucia	2	n	26	132	s	n	n	n	a
f	11	malambo	2	n	59	150	s	n	n	s	ob
m	5	barran-quilla	1	n	22	115	n	n	n	n	a
m	6	barran-quilla	1	n	18	110	s	n	s	n	a
m	6	barran-quilla	0	n	17	1.05	s	s	s	n	a
f	3	soledad	2	s	12.8	1	n	n	s	n	a
m	5	barran-quilla	0	n	18.2	1.09	n	n	s	n	a
f	3	galapa	3	n	11	0.81	s	n	n	n	a
m	15	barran-quilla	1	n	42	1.47	s	n	s	n	a
f	15	sabanalarga	0	s	56	1.6	s	n	s	n	a
f	5	barran-quilla	0	s	14.4	1.05	s	n	s	n	a
m	5	soledad	3	n	27	0.89	s	n	n	n	ob
m	4	malambo	1	s	15	0.94	s	s	s	n	a
f	4	barran-quilla	1	s	19	1.04	s	s	n	s	a
m	3	barran-quillo	1	n	12	0.93	s	n	c	n	a
f	6	barran-quilla	2	n	20	1.13	n	n	n	s	a
f	10	barran-quilla	0	n	32.1	1.49	n	n	n	n	a
f	13	baranoa	1	s	28	1.37	s	n	n	n	a
m	9	barran-quilla	2	n	37	1.4	n	n	n	s	a
f	3	sa-banagrande	1	n	14	0.92	s	s	n	s	a

Continuation **Table S1. Qualitative and quantitative variables.**

Data Emergency Covid-19 period

Gender	Age/years	Geographic	Pulmonary score	Breastfed infants	Body weigth (Kg)	Stature (m)	Hospitalization	Corticosteroids Use	COVID-19 Vaccination	Environmental pollution exposure	Nutritional status
m	5	barranquilla	3	s	18	1.09	s	n	n	s	a
f	7	barranquilla	2	n	22.5	1.18	s	n	n	s	a
f	3	soledad	4	n	9.6	0.72	s	n	n	n	a
m	3	ponedera	2	n	19	1.03	s	n	n	s	a
f	7	barranquilla	1	s	24	1.16	n	n	n	n	a
m	4	malambo	3	n	17	1	n	s	n	n	a
m	4	barranquilla	1	s	18	1.07	n	s	n	n	a
m	7	barranquilla	3	n	34	1.2	n	s	n	s	s
m	4	baranoa	0	s	16.6	1.04	s	s	n	s	a
m	7	santuario	2	s	26	1.27	n	s	n	n	d
f	3	barranquilla	5	n	10.5	0.9	s	s	n	s	a
f	11	barranquilla	3	n	46	1.36	n	n	n	n	s
m	3	barranquilla	2	s	13.5	0.93	n	n	n	s	a
m	6	malambo	3	s	20	1.14	n	n	n	s	a
f	13	ciénega magdalena	2	s	40	1.55	n	n	n	n	a
m	8	galapa	2	s	30	1.25	n	n	n	s	s
m	2	barranquilla	3	s	12.3	0.76	n	s	n	s	s
m	3	barranquilla	1	s	16	0.9	n	n	n	n	s
f	6	la playa	2	s	22	1.22	n	n	n	n	a
f	12	barranquilla	5	n	59	1.5	s	n	n	n	s
m	9	barranquilla	3	n	28	1.35	n	s	n	n	a
m	3	barranquilla	0	n	13.5	0.97	n	s	n	n	a
f	6	barranquilla	2	n	21	1.19	n	s	n	s	a
m	4	barranquilla	3	n	16.5	1.05	n	n	n	s	a

f	3	sa-banagrande	2	n	15.5	1	s	s	n	s	a

Continuation **Table S1. Qualitative and quantitative variables.**

Data Emergency Covid-19 period

Gender	Age/years	Geo-graphic	Pulmo-nary score	Breast-fed in-fants	Body weigth (Kg)	Stature (m)	Hospital-ization	Corticoster-oidsUse	COVID-19 Vac-cination	Environ-mental pollution exposure	Nutri-tional sta-tus
m	5	barran-quilla	3	s	18	1.14	n	s	n	s	a
m	3	soledad	1	s	11.2	0.81	s	s	n	n	a
f	7	barran-quilla	0	n	24	1.25	s	n	s	s	a
m	4	tubará	6	n	17.9	1.09	s	s	s	n	a
m	4	soledad	2	n	11	1	s	s	n	n	a
m	3	barran-quilla	0	n	10.5	0.8	s	n	n	n	a
m	4	barran-quilla	1	n	13.36	0.96	s	s	n	s	a
m	6	soledad	1	s	16	1.05	s	s	s	n	a
f	3	manati	3	n	12.3	92	s	n	n	n	a
f	9	malambo	5	n	29.2	1.42	s	n	s	s	a
m	3	barran-quilla	0	n	17.5	98	s	n	n	n	s
m	6	barran-quilla	4	n	18.8	1.19	s	s	n	n	a
m	5	barran-quilla	4	n	18	1.13	n	s	s	n	a
f	7	barran-quilla	2	n	29.5	1.28	n	s	s	s	s
m	6	saba-nalarga	3	n	17.2	1.14	s	s	n	n	a
f	4	barran-quilla	3	s	12.8	0.93	s	s	n	n	a
f	3	malambo	4	n	9.75	0.77	s	s	n	n	a
m	5	barran-quilla	3	n	16.3	1.1	s	s	n	n	a
m	6	ponedera	4	s	16	1.1	n	n	n	n	a
f	3	moned-era	3	s	10.7	0.8	s	s	n	s	a
f	5	malambo	6	n	13	1	s	n	n	n	d
m	3	barran-quilla	0	s	13.4	0.91	n	n	n	s	a
f	3	ponedera	3	s	13	0.9	s	s	n	n	a
f	5	ponedera	6	n	11	0.9	n	n	n	n	a
m	3	manati	6	n	13	0.9	s	s	n	s	a

Continuation **Table S1. Qualitative and quantitative variables.**

Data Emergency Covid-19 period

Gender	Age/years	Geographic	Pulmonary score	Breast-fed infants	Body weigth (Kg)	Stature (m)	Hospitalization	CorticosteroidsUse	COVID-19 Vaccination	Environmental pollution exposure	Nutritional status
m	3	barranquilla	4	s	14.6	0.98	s	s	n	n	a
m	9	barranquilla	2	n	30	1.4	n	n	n	n	a
f	8	barranquilla	1	s	22.3	1.25	n	n	s	n	a
m	3	soledad	3	n	17.5	0.97	s	n	s	n	s
m	3	soledad	7	n	13.5	0.9	s	s	n	n	a
f	3	ponedera	1	s	12.5	0.95	s	s	n	n	a
f	3	barranquilla	3	s	10.8	0.83	s	s	n	n	a
m	9	barranquilla	1	n	28	1.33	n	n	n	n	a
m	4	puerto colombia	8	n	17	1.14	s	n	n	n	a
f	9	barranquilla	1	s	26	1.24	s	n	s	n	a
f	9	barranquilla	0	s	26	1.24	s	s	n	s	a
f	3	barranquilla	1	n	10	81	s	s	n	n	a
f	3	barranquilla	2	s	9.5	81	s	s	n	n	a
f	3	barranquilla	0	n	8.8	0.75	s	s	n	n	a
m	4	barranquilla	2	n	18	0.95	s	n	n	n	ob
f	4	barranquilla	2	n	15.7	0.95	s	n	s	n	a
m	4	barranquilla	2	n	15	0.96	s	s	s	n	a
m	3	barranquilla	0	n	11.8	0.87	s	s	s	n	a
m	6	barranquilla	0	n	18	1.13	s	s	s	n	a
f	7	barranquilla	0	n	19	1.15	s	s	s	n	a
m	4	barranquilla	0	n	22	1.14	s	s	s	n	a
m	3	barranquilla	0	s	9.84	0.82	s	s	n	s	a
m	5	barranquilla	3	n	12	0.96	s	s	n	s	d
f	7	malambo	2	n	20	1.18	s	s	n	n	a
f	5	soledad	1	n	15	1.05	s	s	s	n	a

Continuation **Table S1. Qualitative and quantitative variables.**

Data Emergency Covid-19 period

Gender	Age/years	Geographic	Pulmonary score	Breastfed infants	Body weigth (Kg)	Stature (m)	Hospitalization	CorticosteroidsUse	COVID-19 Vaccination	Environmental pollution exposure	Nutritional status
m	11	barranquilla	2	s	42	1.45	S	s	n	s	s
f	13	Venezuela	1	s	48	1.47	s	n	n	s	s
f	7	barranquilla	1	s	23	1.2	n	n	n	s	a
m	5	P/Colombia	1	s	19.1	1.13	s	n	n	n	a
f	5	Polo Nuevo	4	n	17.4	1.04	s	s	s	n	a
m	11	barranquilla	1	n	26	1.2	n	n	n	s	a
f	4	Malambo	0	n	12.3	0.9	s	n	s	n	a
m	3	barranquilla	4	s	14	91	s	s	n	n	a
f	7	Polo Nuevo	0	n	20	1.25	s	s	s	s	a
m	12	puerto Colombia	4	n	66	1.3	s	s	s	n	ob
m	4	barranquilla	0	n	16.3	102	s	s	n	n	a
m	7	soledad	2	n	25	1.35	s	s	s	n	a
m	11	barranquilla	2	s	50	1.56	s	s	s	n	s
m	4	barranquilla	0	s	15	97	s	s	s	n	a
f	12	barranquilla	0	s	47.0	1.45	s	s	s	n	s
m	6	soledad	3	s	17.6	1.04	s	s	s	n	s
m	13	barranquilla	3	s	27	1.30	s	s	n	n	a
m	10	Venezuela	5	n	17	1.13	s	s	n	n	d
m	3	barranquilla	3	s	14	0.83	n	n	s	n	s
f	3	barranquilla	3	s	8.7	0.8	n	n	n	n	d
m	5	barranquilla	1	n	12.4	0.8	s	n	n	n	a
f	6	barranquilla	2	s	19	1.15	n	s	s	s	a
f	3	barranquilla	4	s	8.5	0.75	s	s	n	n	a

m	3	barran-quilla	6	n	13	0.84	s	s	n	s	a
m	10	barran-quilla	1	s	28	1.25	s	n	n	n	a

Continuation **Table S1. Qualitative and quantitative variables.**

Data Emergency Covid-19 period

Gender	Age/years	Geographic	Pulmonary score	Breastfed infants	Body weigth (Kg)	Stature (m)	Hospitalization	CorticosteroidsUse	COVID-19 Vaccination	Environmental pollution exposure	Nutritional status
f	4	barranquilla	1	n	13.2	0.87	n	n	n	n	a
f	7	barranquilla	3	s	17.8	1.07	s	n	n	n	a
m	4	barranquilla	2	n	14	0.9	n	n	n	s	a
m	4	barranquilla	1	n	14	1	n	n	n	s	a
f	9	barranquilla	3	n	35	1.3	n	s	n	s	s
f	9	barranquilla	4	n	25	1.28	s	s	n	n	a
m	3	galapa	2	n	14	0.94	s	n	n	s	a
f	5	barranquilla	3	n	16	1.08	s	s	n	n	a
f	8	barranquilla	3	n	39	1.35	n	s	n	s	ob
m	5	barranquilla	2	n	21.5	1.13	s	n	n	n	a
m	4	soledad	2	n	16.5	1.06	s	n	n	n	a
f	4	barranquilla	2	n	16.5	1.07	s	s	n	n	a
f	6	barranquilla	2	n	26	1.2	n	s	n	s	ob
f	7	soledad	1	n	22	1.16	s	n	n	s	a
f	5	Soledad	1	s	16	1.04	s	s	n	n	a
m	3	barranquilla	3	s	14.5	0.99	s	s	n	n	a
f	4	barranquilla	3	s	16.2	104	s	n	n	n	a
f	5	barranquilla	3	n	16	1.08	s	s	n	n	a
f	3	Soledad	3	s	14.5	0.93	s	n	n	n	a
m	5	Soledad	2	n	16	104	s	n	n	n	a
m	7	barranquilla	4	s	32	1.26	s	n	s	n	ob
m	9	barranquilla	3	n	29	1.23	s	n	s	n	s
f	9	barranquilla	3	n	25	1.28	s	n	s	n	a
m	3	barranquilla	2	s	14	0.83	n	n	s	n	s
m	12	barranquilla	2	s	29	1.45	s	s	s	s	a
m	3	soledad	1	n	10.6	79.5	n	s	s	s	a

Continuation **Table S1. Qualitative and quantitative variables.**

Data Emergency post Covid

Gender	Age/years	Geographic	Pulmonary score	Breastfed infants	Body weigth (Kg)	Stature (m)	Hospitalization	CorticosteroidsUse	COVID-19 Vaccination	Environmental pollution exposure	Nutritional status
m	5	malambo	0	n	16.6	0.91	s	s	s	n	s
m	7	galapa	1	s	21	1.2	s	s	s	n	a
m	3	barranquilla	0	s	16.4	1	s	s	s	n	a
m	4	malambo	1	s	19.8	1.09	s	n	s	n	a
m	4	barranquilla	2	s	14	1	s	n	s	n	a
m	4	barranquilla	1	s	18.6	1.13	s	s	n	n	a
f	4	malambo	2	n	14	0.99	s	s	n	n	a
m	4	barranquilla	2	s	16	1.02	n	s	n	s	a
m	9	barranquilla	2	n	28.5	1.35	n	s	s	s	a
m	4	barranquilla	1	s	17.3	1.15	n	n	s	s	a
f	8	barranquilla	3	n	22	1.26	n	n	s	n	a
m	5	barranquilla	1	s	23.1	1.12	s	s	n	n	s
m	15	repelon	2	n	44.5	1.61	s	s	s	n	a
m	3	barranquilla	2	n	13	0.9	s	n	n	n	a
f	12	barranquilla	2	n	35	1.35	n	n	s	n	a
f	12	barranquilla	1	n	35	1.35	n	s	s	n	a
m	4	barranquilla	2	s	17.3	0.98	s	s	n	s	a
f	6	barranquilla	7	n	15	1	s	s	s	s	a
m	10	barranquilla	2	s	46	1.36	n	s	s	n	ob
f	4	soledad	1	s	14.5	0.98	s	n	n	n	a
f	3	barranquilla	3	n	12.3	0.89	n	s	n	n	a
m	4	barranquilla	5	s	9.8	0.86	s	s	s	n	d
m	4	barranquilla	2	s	26	1.1	n	n	n	n	ob

m	8	barran-quilla	2	n	34	1.35	n	s	s	s	s

Continuation **Table S1. Qualitative and quantitative variables.**

Data Emergency post Covid

Gen-der	Age/years	Geographic	Pulmo-nary score	Breast-fed in-fants	Body weigth (Kg)	Stat-ure (m)	Hospitali-zation	Corticoster-oidsUse	COVID-19 Vac-cination	Environ-mental pollution exposure	Nutri-tional sta-tus
m	12	barranquilla	2	s	32	1.5	n	s	s	s	a
m	3	barranquilla	2	n	13.2	0.9	n	s	n	s	a
m	5	soledad	5	n	10	0.85	s	n	s	s	a
m	5	barranquilla	2	n	21	1	s	n	n	n	ob
m	4	malambo	2	s	16.5	1.13	s	s	s	n	a
f	4	barranquilla	2	s	13	0.9	n	s	n	n	a
f	4	barranquilla	1	S	16	1.07	s	s	n	n	a
m	16	Soledad	2	n	30	1.22	s	S	n	s	a
f	4	barranquilla	0	s	15	1.03	s	s	n	n	a
m	4	barranquilla	0	s	16	1.02	n	s	n	s	a
m	3	barranquilla	5	n	18.7	1.1	n	n	n	n	a
m	5	barranquilla	3	s	20.7	1.13	s	s	n	n	a
m	6	malambo	2	s	18	1.12	n	n	n	s	a
m	5	barranquilla	3	n	16.2	105	s	n	n	n	a
m	5	barranquilla	3	s	16	1.06	s	n	n	n	a
f	3	soledad	2	s	13	0.94	s	n	n	n	a
m	4	barranquilla	2	n	20.5	1.04	s	n	n	n	s
m	6	barranquilla	2	n	22.5	1.2	s	n	s	n	a
m	5	barranquilla	2	s	20.7	1.13	s	s	s	n	a
m	4	barranquilla	2	s	16	1.02	n	s	n	s	a
m	4	barranquilla	3	n	16	1.03	n	n	n	s	a

Disclaimer/Publisher's Note: The statements, opinions and data contained in all publications are solely those of the individual author(s) and contributor(s) and not of MDPI and/or the editor(s). MDPI and/or the editor(s) disclaim responsibility for any injury to people or property resulting from any ideas, methods, instructions or products referred to in the content.
